# “*It’s like going through life at a mediocre level*”: a qualitative study of the meaning and impact of fatigue in children and young people with sickle cell disease

**DOI:** 10.1186/s12887-025-05720-7

**Published:** 2025-05-13

**Authors:** Brenda Agyeiwaa Poku, Karl Michael Atkin, John David Grainger, Iyamide Thomas, Rachael Oshinbolu, Abubakar Mohammed, Edith Kyewalyanga, Susan Kirk

**Affiliations:** 1 School of Sociology and Social Policy, Law and Social Science Building, University of Nottingham, Nottingham, NG7 2RD UK; 2 Department of Sociology, University of York, York, UK; 3 School of Medicine, Royal Manchester Children’s Hospital, University of Manchester, Manchester, UK; 4 Sickle Cell Society, London, UK; 5 Lived Experience Contributor, London, UK; 6 School of Health Sciences, University of Manchester, Manchester, UK

**Keywords:** Children, Experiences, Fatigue, Qualitative, Sickle cell disease, Young people

## Abstract

**Background:**

Fatigue is increasingly recognised as a prevalent and debilitating symptom for children and young people (CYP) with long-term conditions (LTCs), significantly affecting their family, social and educational participation. In sickle cell disease (SCD), fatigue is the most frequently reported symptom, surpassing pain related to vaso-occlusion. However, understanding of fatigue’s nature and impact on CYP with SCD remains limited. This qualitative study explores the meaning and consequences of fatigue for CYP with SCD to inform services, treatments and care.

**Methods:**

This exploratory research interviewed 12 CYP with SCD aged 12–23 years, five parents and ten healthcare professionals across England. Participants were recruited through convenience sampling from an NHS Trust, SCD-focused charities and social media. Data were generated using audio-recorded online semi-structured or art-elicitation interviews. Interviews were transcribed and analysed using coding, constant comparison and thematic categorisation to identify key themes.

**Results:**

Six thematic categories were constructed from the data: (1) constant state of reduced energy, (2) the daily struggle, (3) the invisibility of fatigue, (4) being socially isolated, (5) managing fatigue, and (6) the future while negotiating fatigue. SCD fatigue was seen as a persistent, inescapable daily struggle, with reduced energy for day-to-day activities. This was often unnoticed or misunderstood by others. It hindered YP’s daily routines, caused frequent school absences, reduced social participation, and promoted social exclusion. To meet social expectations and avoid stigma, CYP described constantly pushing themselves to conceal their fatigue, exacerbating their difficulties with SCD. Fatigue was invisible in clinical settings, leading to a lack of standardised/formalised support and increasing uncertainties and precarity about the future. CYP and parents employed energy economisation and recharging strategies to cope with and control fatigue.

**Conclusions:**

Fatigue dominates CYP’s experience of living with SCD, significantly impacting their physical, social and educational functioning and leading to isolation and stigma. Often overlooked in clinical settings, addressing fatigue should be integral to SCD care and research. This includes incorporating fatigue assessments, developing targeted self-management programmes, and furthering research on its management. The findings emphasise recognising fatigue as a primary symptom in CYP with LTCs, given its severe impact on social and educational development and future stability.

**Trial registration:**

Not Applicable.

## Background

Fatigue is increasingly recognised as a prevalent and debilitating symptom for children and young people (CYP)^1^ with long-term conditions (LTCs) and those treated for life-threatening illnesses like childhood cancer. Systematic reviews consistently highlight high fatigue prevalence in CYP with LTCs such as juvenile idiopathic arthritis [1], multiple sclerosis [2], and cancer [3], as well as across various chronic conditions [4]. This fatigue, often comparable to chronic fatigue syndrome [5], encompasses physical, cognitive, social and emotional dimensions [1, 6]. Fatigue significantly impacts CYP’s quality of life, well-being, and daily participation—affecting learning, mood, school attendance, and relationships [1–2, 5, 7, 8, 9]. Several studies link fatigue in CYP with LTCs to various biopsychosocial factors, including pain, treatment side effects, low mood, sleep disturbances, and poor school performance [8, 10, 11]. However, fatigue in CYP with LTCs is not associated with disease severity [5, 12, 13] and often persists despite treatment of the underlying condition.

Despite its prevalence and significance, fatigue is often overlooked within interventions like self-management programmes [14] and under-acknowledged by clinicians [15, 16]. Research on fatigue in CYP with LTCs is largely focused on cancer-related fatigue. A recent systematic review on the prevalence of fatigue in CYP aged 0–19 years with LTCs found that studies on paediatric cancer-related fatigue were the most common, comprising 28% of included studies across 12 LTC categories [4]. Notably, research on sickle cell disease (SCD) fatigue is limited.

SCD is the most common genetic haemoglobin disorder, affecting about 300 births annually in the United Kingdom [17] and over 300,000 children globally [18]. The condition can lead to severe complications such as vaso-occlusive crises, recurrent infections, acute chest syndrome, stroke, and multi-organ failure [19]. Patients’ common symptoms include fatigue, bone aches, and headaches, with fatigue frequently reported [20, 21]. A biobehavioural model suggests that factors such as hypoxaemia, chronic haemolytic anaemia, inflammation, pain, stress, depression, and anxiety associated with SCD can cause substantial fatigue [10, 22]. Despite fatigue being a prevalent symptom, there is a glaring lack of data on its prevalence, frequency, severity, and daily life impact. This oversight may be related to the perception that fatigue is inevitable and unresolvable or because SCD affects people of black and ethnic minority backgrounds who are at increased risk of health research disparities [23].

Understanding fatigue in SCD is crucial as it may be both chronic and acute, predict crises [24, 25], and correlate with poorer quality of life [12, 22]. Fatigue is also linked to increased acute pain episodes, depression, anxiety, behavioural difficulties, and higher hospitalisation rates in CYP with SCD [12, 22, 25]. CYP are particularly vulnerable to fatigue and its disabling consequences due to increased activity demands and expectations as they pursue education, relationships, and employment [22]. A recent qualitative study with adolescents with SCD in Ghana found that fatigue in SCD is highly stigmatising, severely disrupting their sense of normalcy and significantly limiting their educational, social, and biographical pursuits [26–28]. Yet fatigue remains underrepresented in self-management interventions for CYP with SCD [29]. Understanding the nature and consequences of fatigue for CYP with SCD is crucial to inform services, treatments and care. This study aimed to explore the perspectives of CYP with SCD, their parents, and health professionals on the meaning and impact of SCD-related fatigue.

## Methods

An exploratory qualitative approach, drawing on the principles of constructivist grounded theory, was employed [30]. The approach acknowledges that knowledge is socially constructed and situated through social interactions and that research data are co-constructed between the researchers and participants. This allowed us to bring our research, clinical and community expertise, knowledge and lived experience of SCD to shape the inquiry while reflecting on how these may influence data collection and analysis [31].

### Recruitment and sampling

We involved three participant groups: CYP with SCD, parents or primary caregivers, and healthcare professionals. Table 1 presents the sample eligibility criteria.

**Table 1:** Sample eligibility criteria

Inclusion Criteria	Exclusion Criteria
• *Children and Young people*: • Aged 12–24 years • Diagnosed with SCD.	• CYP with an additional long-term condition. • CYP who are pregnant • CYP living in foster care.
*Parent*: Mother, father or legal guardian of a young person (12–24 years) with SCD	• CYP/parents assessed by the healthcare team to be inappropriate to include in the study, e.g. recent bereavement, current child protection issues, receiving end-of-life care.
*Healthcare professionals*: Providing services to young people aged 12–24 years with SCD.	• Young people (16 years and above) and parents without the capacity to consent

Eligible participants were recruited from an NHS Trust in North West England and through SCD-focused charities (Sickle Cell Society, OSCAR Sandwell, Sickle Cell Care Manchester, and the Congenital Anaemia Network). Health professionals were recruited through NHS England Haemoglobinopathy Coordinating Centres, the British Psychological Society Special Interest Group in Haemoglobinopathies and the Sickle Cell and Thalassaemia Association of Nurses, Midwives and Allied Health Professionals.

Convenience sampling, supplemented by snowballing, was primarily employed. As the study progressed, purposive sampling was introduced with the aim of including participants with specific characteristics (e.g., male and non-African/Caribbean ethnicity) and from different professional disciplines. Study information was given to eligible participants by clinicians at the Trust and key contacts at the charities and professional networks. Those interested in participating in the study contacted the first author (BAP) to discuss the study further. A time and format for an interview (in-person or remote) were scheduled with those who agreed to participate in the study. During the study recruitment period, BAP also attended monthly clinics at the participating NHS Trust to meet eligible CYP and parent participants who indicated interest in participating. For those under 16 years old, parents were involved in the recruitment process and provided consent for their child’s participation. We aimed to recruit 30 participants (15 CYP, five parents and ten healthcare professionals) for the study. However, theoretical sufficiency was reached with 27 participants.

### Data collection

Data were collected by BAP using semi-structured interviews and art-based approaches. The latter were included to give CYP participants greater flexibility in choosing how they wished to represent and communicate their experiences [32]. Interviews were conducted with 12 CYP, five parents and ten healthcare professionals. All the interviews were conducted between June and November 2022 via videoconferencing (Zoom or MS Teams based on participant’s preference) and lasted between 30 and 120 minutes. During recruitment, CYP participants were invited to engage in art-based activities, such as drawing, photography, poem writing, or creating video diaries to produce artworks related to their fatigue experiences. They selected the preferred activities and were provided with the necessary resources to complete them. These activities were to be completed in advance independently and brought to the interview for discussion. Seven CYP expressed interest in art-based methods and produced artworks. Four produced drawings and/or photographs, two produced poems and one produced a video diary. These artworks were used to stimulate and facilitate the interviews and data generation. Topic guides were developed for each participant group to guide the interviews. Table 2 presents the main topic areas in the topic guides. The guides and interviews focused on participants’ perspectives on the meaning and impact of SCD-related fatigue in CYP; the strategies and resources used by CYP and parents to self-manage fatigue; and current service provision for SCD-related fatigue and potential facilitators and barriers to fatigue management in routine care. Informed consent and assent (from those under 16) were obtained from all participants before participation in data collection.

Four CYP-parent dyads participated in the study, and the child and parent were interviewed separately in each instance. All interviews were audio-recorded and conducted in English. Although provision was made for interpreters where needed, all the CYP and parent participants chose to have their interviews in English and were competent in conversational English. With participants’ permission, interviews were audio-recorded and professionally transcribed. Consent was sought to use the interview excerpts and artworks anonymously during research dissemination. Participating CYP and parents were offered a £20 gift voucher as a token of appreciation.

**Table 2:** Interview topic guides

Participant group	Main topic areas
**CYP**	1. Experience of living with SCD in general a. Daily life with SCD and severity of condition (symptoms, hospital stays) b. Impact on daily life (physical, social, educational/academic, emotional/mental, employment) c. Influence on relationships– parents, siblings, peers, intimate partners d. Self-management– practices, challenges/difficulties, challenges in different contexts (home, school, work, other) e. Support (sources, types, helpful/unhelpful) and support needs 2. Experience of fatigue a. Nature of fatigue– description, explanation of feeling of fatigue b. Impact of fatigue– physical, social, educational/academic, emotional/mental, employment c. Factors influencing fatigue– contributory factors, alleviating factors, contextual (personal, family, social, educational, employment) d. Concerns and worries about fatigue 3. Self-management of fatigue a. Day-to-day self-management– practices, experiences and concerns b. Challenges in different environments– home, hospital, school, work, other c. Views about current support (forms, sources, helpful/unhelpful) d. Support needs and perceptions of unmet support needs
**Parents**	1. Experience of SCD in general a. Severity of child’s condition (symptoms, hospital stays) b. Impact of SCD on child’s and family’s life 2. Management of child’s conditions a. Practices and strategies b. Concerns about managing child’s condition c. Challenges/difficulties in different contexts (home, school, work) d. Support (sources, types, helpful/unhelpful), support needs (perceptions and concerns) e. Perceptions of child’s self-management skills f. Views about transferring management responsibilities to the child g. Experiences and concerns regarding role transfer 3. Child’s fatigue experience a. Perceptions of the nature of child’s fatigue– description of child’s fatigue b. Impact of child’s fatigue on child and family’s life c. Factors influencing child’s fatigue d. Concerns and worries about child’s fatigue e. Strategies for managing child’s fatigue f. Experiences of providing support g. Experiences of healthcare provision for child’s fatigue 4. Management of fatigue a. Day-to-day self-management– practices, experiences, concerns b. Challenges in different environments– home, school, work c. Views about current support (forms, sources, helpful/unhelpful) d. Support needs and perceptions of unmet needs
**Healthcare professionals**	1. SCD care in general a. Organisation of services and standards of care b. Current treatments and support programmes c. Views and concerns about current care 2. Self-management among CYP a. Preparation of CYP for self-management b. Current programmes used to support self-management c. Barriers/facilitators to providing self-management support 3. Perspectives on SCD fatigue a. Causes- biological, psychological, social b. Influencing factors– contributing and alleviating factors c. Impact of fatigue on CYP d. Vies and concerns about fatigue in CYP 4. Fatigue management a. Current treatment/support programmes for fatigue b. Experiences of providing fatigue support to CYP and families c. Barriers/facilitators to providing fatigue support and self-management interventions

### Data analysis

Interview transcripts and artworks were anonymised^2^ and transcripts were imported into NVivo 12 Pro to organise and manage the data. Using the constructivist grounded theory approach [30], data were analysed iteratively through coding, constant comparisons, thematic categorisation and concurrent data collection and analysis. BAP coded the transcripts. BAP, KMA and SK met regularly to discuss the emerging codes, category development and interpretation. Coding and category development guided subsequent data collection. Regular meetings among the authors were also used to reflect on the data and developing themes, seeking alternative and possible themes and interpretations. This helped to increase the credibility of the analysis and identify how our experiences and insights about the research area might influence data analysis and interpretation. The reporting of the findings below has prioritised the CYP accounts and used the experiences of parents and healthcare professionals as a commentary on the CYP’s accounts. The analysis generated six thematic categories: *a constant state of reduced energy*, *the daily struggle*, *the invisibility of fatigue*, *being socially isolated*, *managing fatigue*, and *the future while negotiating fatigue*.

### Ethics

Ethical approval for the study was received from the UK’s Health Research Authority (REC reference: 22/SW/0036; IRAS ID: 310855) before recruitment. All study participants were provided with a tailored participant information sheet. Audio-recorded verbal consent and/or assent was obtained before the start of every interview. For CYP under 16 years, consent was obtained from parents before they were approached about participation in the study. Parents also provided proxy consent before their children were interviewed. If the CYP did not give assent, they were not asked to participate in the interview, even if their parents consented to their participation. Consent was regarded as a continual process, with attention paid to any indication that participants might wish to discontinue the interview. Participants were fully informed about the purpose of the art-based methods, the potential copyright and anonymity issues that might arise, and their consent and/or assent were sought for the use of the artwork for data generation and research dissemination. As there was a risk that participants might become distressed during the interviews, a distress and debrief policy was developed to ensure participants were supported both during and after participation in the study. Procedures were also established for any safeguarding disclosures, and confidentiality limitations were highlighted in the participant information sheets.

### Public and patient involvement

We involved three young people with SCD (co-authors RO, AM and EK) aged 18–24 years as project advisors who contributed to various stages of the research. Notably, two (EK and RO) were actively involved in developing the grant application, shaping the research from its early stages. The young advisors were also instrumental in developing participant-facing recruitment materials– study information sheets, consent/assent forms for CYP participants and a recruitment video. They contributed to developing topic guides for data collection and were involved in data analysis, interpretation and dissemination.

## Results

The sample for our study comprised 12 CYP, five parents (all mothers), and 10 healthcare professionals from various disciplines (Table 3). The CYP ranged in age from 12 to 23 years old, with five aged between 12 and 15 years old and seven between 16 and 23 years old. Eight CYP identified as female and four as male. It is worth noting that there were no CYP or parent participants from a non-African/Caribbean background, which is not unusual as SCD predominantly affects people of African and Afro-Caribbean backgrounds in the UK [33].

**Table 3:** Participant characteristics

CYP characteristics	Number
Number of participants	**12**
Sex	
Male Female	4 8
Ethnicity	
Black-British (African) Black-British (Caribbean) Black African Mixed (White & African)	5 2 4 1
Age	
12–15 years 16–23 years	5 7
SCD diagnosis	
HbSS HbSC	10 2
Hydroxycarbamide Exchange blood transfusion	
Hydroxycarbamide Exchange blood transfusion	8 2
Mild Moderate Severe	
Mild Moderate Severe	2 8 2
Current Educational level	
Primary School Secondary School College Postgraduate	4 1 6 1
Parent characteristics	**Number**
Number	**5**
Sex	5
Female	5
Ethnicity	
Black-British (African) Black-British (Caribbean) Black African Black Caribbean	1 1 2 1
Child’s SCD diagnosis	
HbSS HbS-Beta Thalassaemia	4 1
Perceived severity of child’s SCD	
Mild Moderate Severe	1 3 1
Educational level (Child)	
Primary Secondary Special Education	3 1 1
HCP characteristics	**Number**
Number of participants	**10**
Discipline/Job title	
Consultant haematologist Specialist nurse (hospital care) Specialist nurse (community care) Clinical psychologist Specialist physiotherapist Youth worker	1 3 1 3 1 1

The findings are presented around the six categories generated from the analysis. The categories present an overall line of argument about how fatigue is experienced as a constant daily struggle of reduced energy, which is invisible to others, promotes social exclusion, is difficult to manage, and creates uncertainties about the future.

### A constant state of reduced energy

Across all the interviews, participants clearly described fatigue and how it influenced their everyday lives. They described their fatigue using metaphors related to low energy:

It’s like having the appearance of a Land Rover but the energy capacity of a small Fiat (CYP10, male, aged 16 years).

It’s like going through life constantly on a low battery (CYP08, male, aged 12 years).

**Fig. 1:**
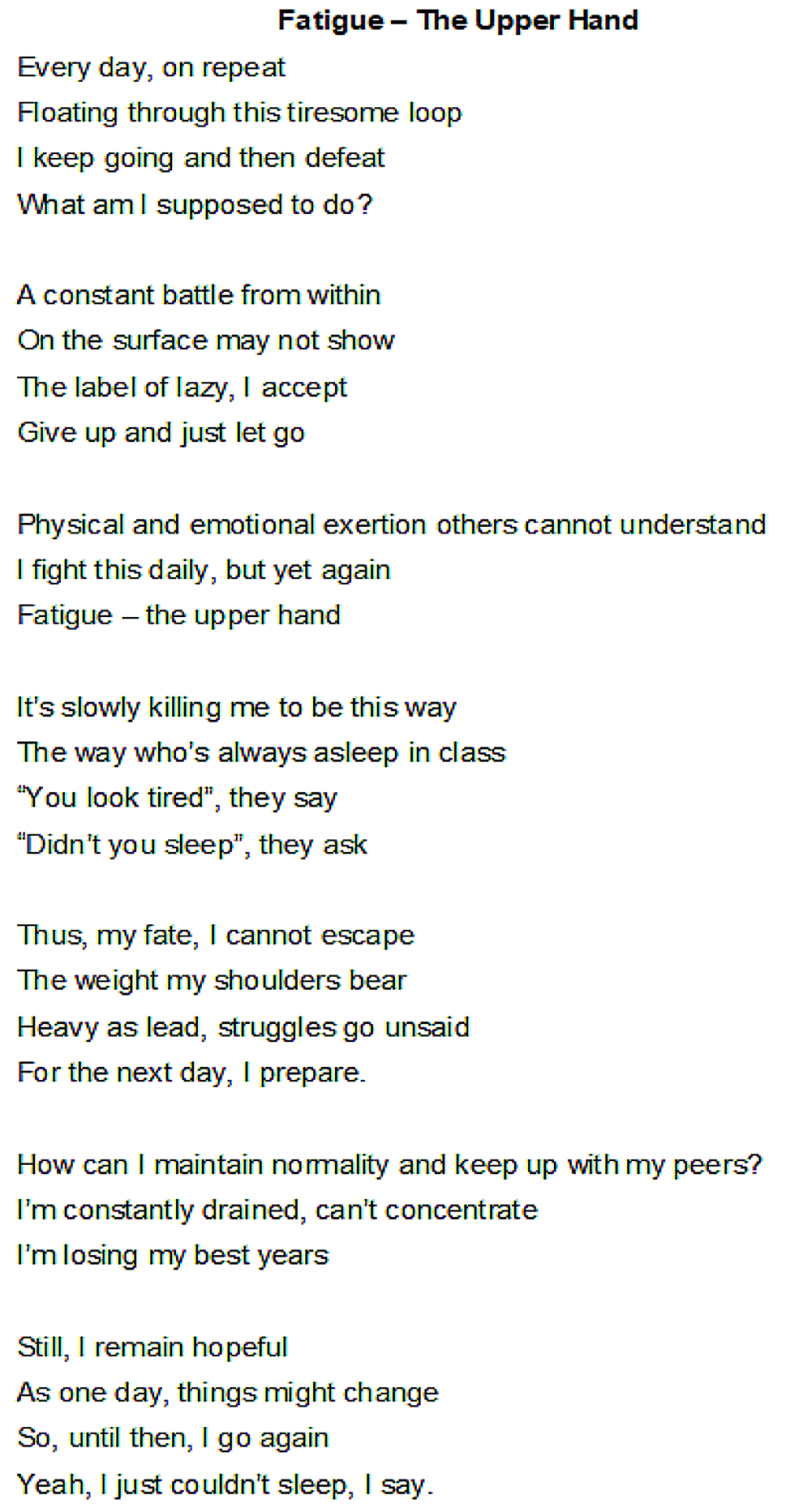
Poem by CYP02, female, aged 23 years

CYP regarded fatigue as a fundamental feature of SCD and, therefore, inescapable, with one young person emphasising how it was, *thus, my fate, I cannot escape* (Fig. 1, poem by CYP02, female, aged 23 years). They identified biological factors as being the underlying cause of SCD fatigue, using terms such as *low haemoglobin, chronic anaemia, sickled red cells*, and *hypoxaemia*. Therefore, SCD fatigue was externalised as something they had no control over. However, they were aware that their fatigue was exacerbated by dehydration, exertion (physical, social, emotional, cognitive or mental), pain crises, strong pain medications, not taking SCD medication on time, and not eating well. CYP associated fatigue with feelings of exhaustion, dizziness, shortness of breath, forgetfulness, concentration difficulties and an overwhelming urge to sleep. Indeed, for CYP, there appeared to be a close and intertwined relationship between fatigue and SCD pain, with fatigue being described as a trigger, a consequence and a warning sign of an imminent pain crisis. This caused anxiety as the CYP felt they could not always mitigate the risk.

SCD fatigue was described as being present even at rest, and CYP felt that they never had sufficient energy for day-to-day activities. Fatigue was characterised as constant and dynamic, with CYP *moving between a state of low energy and a state of no energy* (CYP02, female, 23 years). SCD fatigue was seen as unpredictable and often not proportional to recent activity, which meant that what CYP could tolerate energy-wise varied from day to day. They found it difficult to make plans and commitments, as they were unsure they could see them through. This created uncertainties and added to the precarity created by having a long-standing chronic condition (this is further discussed below). Fatigue appeared to increase in frequency, severity and impact as CYP transitioned through educational and social stages, took on more responsibilities, and pursued interests and opportunities.

### The daily struggle

SCD fatigue was experienced as a physical and mental ‘heaviness’ that presented CYP with a daily struggle to navigate and overcome. CYP described it as *having your bones replaced with lead* (CYP11, female, 16 years), *wearing a puffer coat filled with rocks* (CYP06, female, 18 years) *and a big grey cloud that sits over you* (CYP12, female, 14 years). Consequently, extreme mental and physical effort was needed for daily activities such as getting out of bed in the morning, keeping their eyes open and remaining attentive in school. CYP07 explained the constant physical and mental struggle:

With sickle cell anaemia, there’s the constant physical agony of fatigue we go through and getting up is difficult…not only is it a physical struggle, but it’s also a mental struggle… having to gain the strength to get up. Like it’s a constant, oh, how am I going to get up now from this exhaustion? Or where do I get the strength to get up from my bed every morning? (CYP07, female, aged 20 years)

The representation of SCD fatigue as a daily struggle likened the CYP’s lives to *being in a war zone, where they had to fight against the odds* (CYP02, female, 23 years). They described how they were involved in a daily fight against their fatigue (and themselves) to engage in daily life. For instance, they described fighting to stay awake to concentrate in school, a fight they mostly lost. This daily struggle set SCD fatigue apart from ‘normal’ tiredness, which, as expressed in CYP11’s account below, was described as a transient experience associated with extreme exertion and relieved by sleep/rest. The CYP were aware that their reduced energy and the daily struggle were different from that of peers:

My fatigue is present when I wake up, present throughout the day, and present when I go to sleep. It can also come alongside a pain crisis. I just ignore it and push through it every day. It’s not like normal tiredness that people normally experience, which happens after a long day or intense exercise and is relieved after sleeping (CYP11, female, aged 16 years).

CYP described pushing themselves physically and mentally to navigate and overcome the daily struggle, as seen in CYP11 and CYP07’s accounts above. They explained how this was an important skill for mastering everyday school, social and family activities that were limited by fatigue in order to maintain a sense of ‘normality’ even though they were aware that ignoring and pushing through came with the risk of triggering a pain crisis. This notwithstanding, fatigue was seen as always having the *upper hand*; while CYP could feel trapped in this fight, they persisted, hoping the next day would be better. One young person extensively captured this sense of battling fatigue in a poignant poem (Fig. 1, poem by CYP02, female, aged 23 years).

The school was particularly singled out as the site of a *constant battle* with fatigue (see Fig. 1). CYP reported frequent school absences, non-participation or reduced participation in schoolwork, and overwhelming tiredness, leading to sleeping in class. Consequently, CYP found it difficult to ‘keep up’ with school/college work, which could negatively impact their educational achievements. As CYP10 explained:

It has sometimes made it difficult to concentrate in class. And last year, this was happening a lot. I would just get home and sleep when I had a lot of schoolwork to do, but I couldn’t stay awake at the end of the day. So, I started to fall behind a little bit, so I had to spend the whole summer just trying to catch up. (CYP10, male, aged 16 years).

The unpredictability, inevitability, constant battle, and pervasive nature of SCD fatigue meant that participants had normalised its presence. There was a sense of resigned acceptance for the CYP, with one child alluding *that is how it is with sickle cell* (CYP05, male, aged 14 years). The normalisation of SCD fatigue contributed to its invisibility to others. However, for several of the parents, the inevitability and invisibility of SCD fatigue led to feelings of disempowerment and a sense of helplessness– this is further discussed below.

### The invisibility of fatigue

Maintaining the invisibility of fatigue was perceived as important by CYP in order to ‘pass’ as ‘healthy’ and ‘normal’ within their social settings. CYP described aiming to achieve this by *pushing through the daily struggle* and *hoping no one sees* (CYP11, female, 16 years) their struggle with fatigue. However, their efforts to hide and mask SCD fatigue were not always successful, which could ‘out’ CYP, raising suspicions about their normality and exposing them to stigma. According to CYP, SCD fatigue betrayed their daily efforts to prevent their illness from being their identity.

Indeed, SCD fatigue was seen as a discreditable attribute and an enduring identity feature for CYP with SCD. In particular, others could not distinguish SCD fatigue from ‘normal’ tiredness and thus failed to appreciate CYP’s daily struggle.

When you tell people you are tired, they think it’s normal tiredness, and when you tell them it’s not, they say, okay, so what have you done today? And you narrate to them that, oh, I’ve just been here and there, just gone to this place because I have to do this today or I have to do that today… to them, that is not supposed to make you tired because it’s not stressful when you tell them about it. But they don’t realise that the energy you need to use to power yourself is double what they would use to do one activity, and this is energy that you normally don’t have too (CYP01, female, aged 20 years).

Having their explanations for their lack of energy discredited and contested by others forced CYP into silence and to accept the label of *lazy*. This further supported their strategy of masking their fatigue, as CYP10 explained:

I’ve become quite good at making the fatigue less noticeable, even if it’s there. I don’t want to bring it up, it gets frustrating from all the interrogations. Because I think to them it looks like me not wanting to do much. I guess it looks like being lazy or just putting in no effort. And I could tell them what’s going on, but I think it would just be a lot easier for me to keep quiet and go on with things (CYP10, male, 17 years).

The daily struggle with reduced energy levels and the stigmatising responses to their fatigue highlight the precarious situation that fatigue creates for CYP. To maintain a sense of normalcy, CYP often push themselves to function despite their fatigue, striving to keep their struggle invisible to others, as CYP11 presents in her poem:

You want to lie down, wait for it to pass

But life waits for no one

So, you push through, knowing your body will push back

But you do it anyway

Hoping no one sees

The awfulness of fatigue

(excerpt from a poem by CYP11, female, aged 16 years)

This effort requires a persistent approach, as they must continuously manage their energy levels and maintain ‘normal’ outward appearances. The need to conceal fatigue and maintain normalcy underlines a form of resilience that CYP develop. This resilience emerges as a response to the vulnerability, uncertainty, and precarity that fatigue creates, as CYP navigate these challenges while seeking to uphold their daily routines and responsibilities.

CYP felt that SCD fatigue was invisible to teachers, healthcare professionals, and services. According to CYP and parents, it was not considered in routine health assessments, patient/parent education, transition services/support, and health consultations. Indeed, for most of the CYP and parents, the interviews were the first time they had talked about SCD fatigue:

I have never talked about it with them [healthcare professionals] because they don’t ask about it. I don’t know if there’s anything they can do about it (P05, child aged 15 years).

They just ask do you get tired at school. And obviously I say yes and then they’re like, next question, let’s measure you, let’s do this. (CYP06, female, aged 18 years)

Rather, CYP and their parents said SCD care and service provision appeared to focus on SCD pain management and monitoring/managing complications. As a result, CYP and their families normalised and minimised the symptom and rarely raised it during healthcare consultations even when it significantly impacted on CYP’s quality of life. This created a sense that fatigue was an unimportant and unresolvable problem that families had to cope with alone, disempowering CYP and parents in seeking help for fatigue.

From healthcare professionals’ accounts, the invisibility of SCD fatigue appeared to relate to there being a limited evidence-based understanding of the symptom, a lack of evidence-based interventions, and the absence of validated fatigue assessment tools for SCD.

I think that we don’t realise how important fatigue is. And I think we don’t have an understanding of just how important it is. If you ask a haematology doctor about sickle cell, fatigue would be one of the things that they mention when they talk about it. But I think there’s no way to measure it, and I think that is what we’re not very good at. When we can’t measure something physically, it’s harder to quantify, and it relies more on qualitative conversation.…. we tend not to give it enough airtime, it’s not even thought of. (HCP05, Clinical Psychologist)

Indeed, some healthcare professionals struggled to explain SCD fatigue, sometimes conflating it with ‘normal’ tiredness or associating it with supposedly typical teenage behaviours of *being lazy, being bored*, and *having poor sleep etiquette*. As HCP05 suggests above, the subjective nature of fatigue means that it can be perceived as unmedical and, therefore, an untreatable symptom. Indeed, many healthcare professionals reported feeling unequipped and unconfident in supporting CYP with this symptom:

It’s the one symptom you know is present, but you don’t ask a lot about it because you don’t know what support to provide (HCP10, haematology consultant).

If a child said in a clinic appointment that they were really struggling to manage their tiredness or fatigue, I don’t know where that would lead to. I don’t know if we have a pathway in the same way that we have a pathway for pain management or recovering from a stroke. (HCP05, Clinical Psychologist)

It was notable that when fatigue had been discussed during a consultation, CYP had found it helpful even without an ‘objective’ fatigue assessment, as it validated their experiences and acknowledged that fatigue was a significant part of their lives with SCD. Indeed, the opportunity to discuss fatigue during consultations was identified as fundamental to bringing legitimacy to SCD fatigue and as a helpful intervention in its own right.

Several CYP shared insights into how healthcare professionals could better incorporate fatigue into consultations. They noted that conversations focused on day-to-day activities, such as managing school and friendships, often provided a natural entry point to discuss fatigue. For instance, one CYP shared how a simple inquiry about school transitions led to helpful advice:

I have had advice from my consultant about it [my condition], but most of it is about managing and stuff, like preventing from going into crisis. I have brought it [my fatigue and energy] up with my consultant. It only happened once when I brought it up. They don’t offer specific support for fatigue; I haven’t seen that kind of support. I think I had started Sixth form, and they [the consultant] asked me how I was getting to and from school, and I think I was saying how it was making me feel very tired. I think that’s how I brought the issue of my fatigue up first. The advice was to leave home earlier and take things a bit slower. That advice has been very helpful. (CYP10, male, aged 17 years)

This account highlights the value of using key transitions in CYP’s social and educational milestones as opportunities to discuss fatigue. Healthcare professionals need to recognise the impact of SCD on CYP’s daily lives and development and tailor their communication to explore these challenges.

Another CYP suggested specific questions that could facilitate meaningful discussions and assessment of fatigue:

Like the questions you’re asking me. What I’m able to do, what I’m not able to do and what I’ll like to do because of my tiredness. How I feel when I’m tired and how people at school and at home treat my tiredness. That way, they can understand it and probably see how they can help. (CYP08, male, 12 years old)

Direct inquiries about fatigue in health consultations would move it from an invisible symptom to the foreground to promote understanding and support. This would enable healthcare professionals to provide simple yet meaningful support to improve care for CYP with SCD in the absence of robust intervention.

### Being socially isolated

**Fig. 2:**
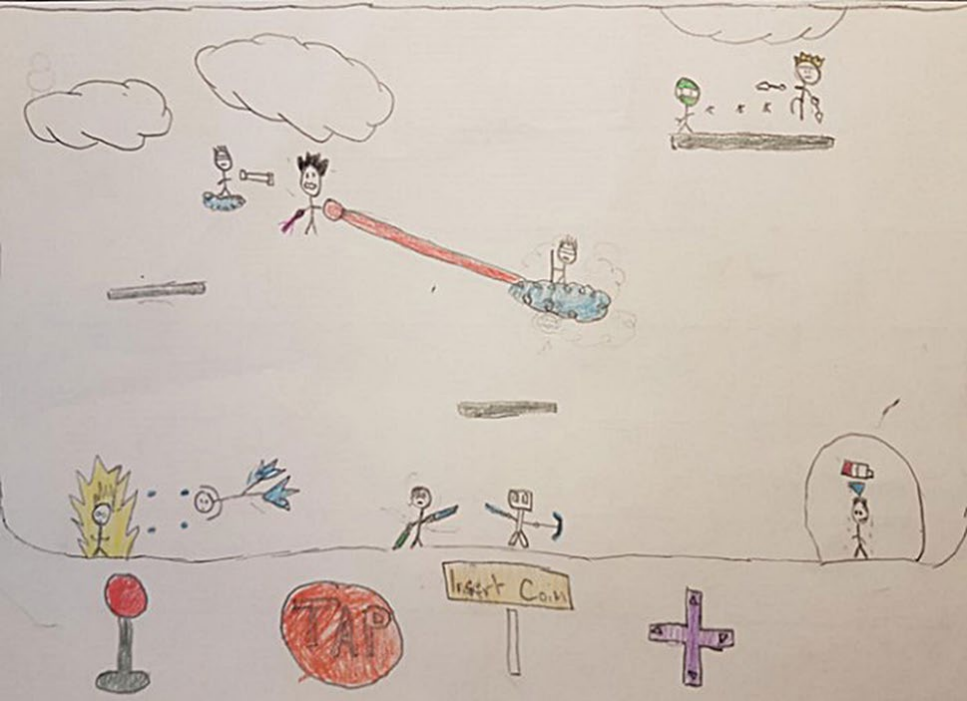
Drawing by CYP04

Fatigue was found to influence CYP’s ability to engage in social activities and to develop and maintain social relationships. During interactions with peers, CYP described how their fatigue made them feel being *in a bubble* and separate from their peers, as CYP04 conveyed in a drawing (see Fig. 2):

*This drawing is about me in a video game, and I’m separated from everyone else. I can’t like play because I’m tired and yeah, and I’m low in energy. I’m that figure in the circle, which is like a bubble. Everyone else is kind of doing something in the game except me* (CYP04, male, aged 12 years).

This sense of detachment and CYP’s tendency to regularly cancel social plans could be interpreted by their peers as the CYP having an attitude problem and being unreliable:

Socially, I’m the friend that’s always tired, and I cancel plans a lot if I’m just tired. Or if I’m already out, or I’m surrounded by my friends, or whatever, I’m just, sometimes I’m just quiet, and literally don’t sometimes have the energy to speak. Like I can be observing what’s going on, my friends might think I’m in a mood or something because I don’t really respond. But I’m literally just in my own world because I’m so tired. And then, I’m there, but I’m not really there: I’m kind of like a blank canvas (CYP02, female, aged 23 years).

On days when I’m struggling with fatigue, say I’m organising a day out with my friends, and at the last minute, I have to cancel. I think things upset me because this time and age, I really want to experience life. Just have fun and do normal teenage things, but that is not always possible. (CYP11, female, aged 16 years)

**Fig. 3:**
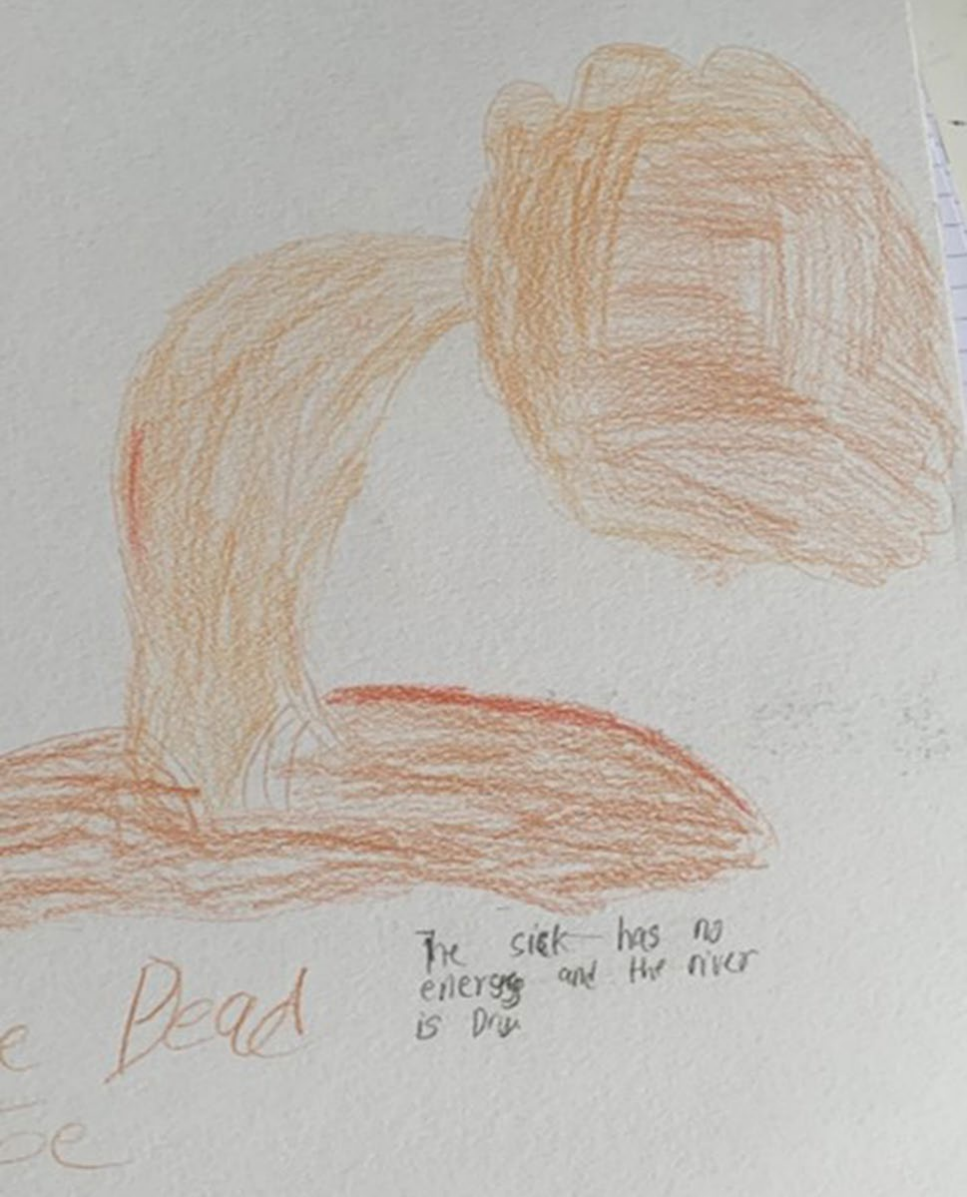
Drawing by CYP08

Consequently, bringing their authentic selves to social relationships and interactions was challenging as they struggled to sustain the expected level of engagement. Fatigue creates a barrier between CYP and what they want to do, encouraging social isolation and withdrawal and a preference for solitary activities as CYP found it exhausting to interact with others and engage in social activities. This made them feel they were *losing their best years* (CYP02, see Fig. 1) as they struggled to *experience life* and *do normal teenage things* (CYP11, female, 16 years). One CYP described life with SCD fatigue as like being *a dead tree* (i.e., being socially dead) in their drawing (see Fig. 3).

The tiredness makes me feel like a dead tree, like there’s no life in me because I can’t do a lot of things, and I’m mostly on my own. There’s no sparkle in my life; it’s just dull and boring (CYP08, male, aged 12 years).

Parents felt that SCD fatigue limited their ability to encourage and support their child’s social participation and independence:

It’s hard to even get him to go out and be with his friends or engage with people. He says, ‘mummy, you don’t understand, I’m tired’. It’s hard for me to see him struggle in that way. He’s at the age he should be physically and socially active, and he just can’t because of the fatigue (P01, child aged 14 years).

The difficulties CYP experienced in social participation due to fatigue led them and their parents to describe feelings of guilt, fear, worry, anger, sadness, frustration and despair. As will be discussed later, participants were concerned about the future implications of social exclusion for CYP’s future.

### Managing fatigue

In the absence of service provision for SCD fatigue, CYP and parents relied on their own creativity and ingenuity to manage the symptom. Participants described using a range of self-management strategies informed by everyday experience and ‘common sense’ that centered around energy management (i.e., energy preservation and ‘recharging’). These self-management strategies had variable ‘effectiveness’ and included rest/sleep, activity pacing, hydration, multivitamin supplementation, physical exercise, solitude/solitary activities and healthy eating. As one CYP explained:

For me, drinking a lot of water, eating well and doing calm things on my own is how I deal with fatigue, like the way you charge a phone, an electric car and water a flower (CYP08, male, aged 12 years).

Self-management was the CYP’s way of asserting control over fatigue and their lives. It involved CYP making daily decisions on how to effectively use their energy, which involved prioritising activities and planning ahead. When making these decisions, CYP evaluated potential activities in terms of their costs and benefits and the potential energy consequences of their actions. This required strict self-regulation, self-discipline, and stringent prioritisation of energy expenditure. As one participant explained, it also involved CYP coming to understand their own bodies and personal limits:

I recommend just knowing your limits and knowing how to get rest. Because when you go over your limit, you’ll usually notice because that’s when the severe fatigue and tiredness will kick in. When I didn’t understand my body and fatigue, my fatigue was pretty bad; I felt tired all the time until I understood what was happening in my body and how to deal with it. Now, I keep track of everything I do, and that has helped me to come to know my body. At first, I just kept pushing myself because back then, I didn’t know my limits, so I was pushing myself even more, which caused me to be tired when I wasn’t even doing anything. Because then in my body, I just felt tired all the time, and I felt lightheaded, and I always had to sleep (CYP05, male, aged 14 years).

In relation to rest, CYP described having to balance the need to be ‘productive’ whilst making time for rest to avoid overexertion and the risk of triggering a crisis. This requires them to make calculations different to those of their peers, which further contributes to their sense of difference:

The struggle is moderating how much rest I take and balancing it with what I have to do. Sometimes, I’ll be like resting now, and then I rest a bit too much, and then I’ll have to rush to get on top of my work and get the work done. So, it’s finding the balance between making sure I have enough energy but not taking too long with the rest. That’s hard (CYP10, male, aged 16 years).

It’s difficult to find the balance between being productive and not overexerting yourself. When your baseline energy is biologically low, you seem to overexert even when you’re doing less (CYP11, female, aged 16 years).

The wider social context influenced the use of fatigue management strategies. For instance, family finances influenced the ability of CYP to eat healthily, engage in exercise (e.g., gym membership), access solitary and less energy-demanding activities (e.g., art/music programmes), and purchase multivitamin supplements and energy-boosting fluids. Family housing circumstances could restrict the availability of comfortable spaces to promote quality rest/sleep. In addition, the demands of life and sociocultural expectations created barriers to using rest and pacing as strategies. CYP perceived these strategies as unrealistic for their day-to-day lives, particularly in school/college, due to inflexible routines, lack of conducive rest spaces and the need to ‘keep up’ with schoolwork:

It’s not everywhere that you can rest and pace yourself. And you can’t do it all the time, too. If I have a wave of fatigue that hits me while I’m at school or something, there’s not really a lot I can do to rest. I can’t just stop everything I’m doing. So, I just try and continue with what I’m doing, knowing that when I get home, that could impact me feeling worse the next couple of days or even with some sort of pain crisis (CYP06, female, aged 18 years).

Indeed, rest and pacing were perceived to be antithetical to youthfulness and productivity. As one young person explained, rest and pacing could have a detrimental effect on their education, employment, finances and social life:

That advice of rest and pace yourself is given without consideration for how realistic they are. You can’t just rest and pace yourself without detriment to your education, employment, finances or social life. I just shake my head when they tell me that because life doesn’t wait for you to rest. Resting and pacing means you’re always playing catch up or missing out or going through life at a mediocre level (CYP02, female, aged 23 years).

### The future while negotiating fatigue

SCD fatigue created significant fear, concern, and uncertainty amongst CYP and parents over whether CYP would have the necessary energy and physical capacity to assume adult roles and be ‘successful’ independent adults. The CYP and parents highlighted similar concerns. However, the CYP appeared calmer when talking about them, while the parents seemed distressed. The lack of attention given to fatigue in clinical care exacerbated CYP’s and parents’ fears, concerns, and anxieties for the future.

I am worried about when I get older. How will I cope when I start having a family of my own? I’m already struggling with work due to the fatigue (CYP02, female, aged 23 years).

I wish it would stop. He’s going to have to go through puberty, and I don’t know where he’s going to get the energy from. I’m afraid he will struggle in life and not have the energy and the strength to do the things every child is supposed to as they grow (P03, child aged 12 years).

These concerns contributed to the CYP’s sense of precarity and uncertainty. Fatigue was seen as reducing CYP’s capacity to build the personal, social and educational capital for adulthood as it impaired their social, educational and physical functioning during childhood and adolescence. The disruptive nature of fatigue required CYP to consider its future consequences, which required negotiation. In terms of future career plans, fatigue had forced some young people to alter these in order to find a career that would fit a future with constant fatigue. CYP06 explained how she had been forced to abandon her career aspirations of being a psychologist and instead pursue a business apprenticeship due to SCD fatigue:

I’m going to go to college to do an apprenticeship. I chose an apprenticeship instead of uni because of the fatigue. The apprenticeship will be a bit more manageable for me. It works for me as an individual rather than what I want to really do in a career. I’d have studied psychology at uni if I was going. So, fatigue affects my decisions about what I want to do in the future. (CYP06, female, aged 18 years)

Healthcare professionals were similarly concerned about the future of CYP. One haematology consultant highlighted their concerns about how fatigue could limit CYP’s future economic potential and independence due to their difficulties in taking up opportunities during adolescence that build economic and social capital:

Fatigue is going to limit their economic potential. These young people are struggling at school due to fatigue. I’m worried that they are going to grow up trapped in the economic deprivation and hardship most are already growing up under. How are they going to attain true independence? This will significantly affect their health in adulthood, increasing the burden on the health services. Due to fatigue, they can’t take advantage of many opportunities and roles necessary for their development. (HCP10, Haematology Consultant)

## Discussion

This study offers comprehensive insights into SCD-related fatigue in CYP with SCD, drawing from the perspectives of CYP, parents and healthcare professionals, thereby contributing to the currently limited understanding of fatigue in SCD. The findings illuminated how fatigue shapes the lives of CYP with SCD, portraying it as a pervasive, unpredictable and omnipresent symptom that significantly impacts their daily functioning across physical, social and educational domains. CYP vividly described fatigue as an ongoing battle with exhaustion, comparing it to carrying burdensome weights like having bones replaced with lead and wearing a coat filled with rocks, which hinders basic tasks such as waking up and staying attentive in school. These descriptors echo similar themes found in studies involving adolescents with chronic conditions such as multiple sclerosis and chronic fatigue syndrome [5, 34]. The school was singled out as the site of the battle with fatigue, causing frequent school absences, reduced participation in schoolwork and activities, and sleeping during lessons.

The distinction between SCD fatigue and normal tiredness was underscored by CYP’s accounts, emphasising the constant presence and debilitating nature of SCD fatigue throughout the day. Despite this, peers and teachers often fail to recognise the severity of CYP’s fatigue, leading to their experiences being contested, discredited, or labelled laziness, even when they try to explain it. Consequently, CYP feel compelled to conceal and mask their fatigue, pushing themselves beyond their limits to conform to societal expectations, as seen in other chronic illness contexts [2, 26, 34–36]. Throughout the CYP’s accounts, there seems to be little room for them to contest expectations and demands posed by others (and themselves) on their level of performance and engagement. If they resist or fail to assimilate, they risk developing a stigmatising sense of personal laziness and irresponsibility [26, 27, 36].

A unique contribution of this study is the revelation of the invisibility of SCD fatigue within clinical care settings. Healthcare professionals appear to often overlook or minimise discussions of fatigue during routine assessment and consultations due to the lack of standardised assessment tools and treatment protocols and pathways, coupled with over-normalisation and a broader misunderstanding that conflates SCD fatigue with everyday tiredness or typical adolescent behaviours (e.g., the propensity to sleep in, reticence to engage in activities). These issues sidelined fatigue as a less prioritised concern. The oversight perpetuates the normalisation of fatigue among CYP and parents, fostering a sense of disempowerment in seeking appropriate support, consistent with findings in studies of chronic fatigue syndrome [34, 37]. However, where health professionals did acknowledge and discuss fatigue with CYP, it validated their experiences and provided a crucial opportunity for supportive intervention. This highlights the importance of integrating discussions about fatigue into routine care to enhance understanding and support the quality of life for CYP with SCD.

The theme of *being socially isolated* highlighted how SCD fatigue impedes CYP’s social participation and relationships, resulting in the cancellation of social plans, forsaking cherished activities and diminished ability to engage authentically with peers. CYP described feeling detached and often misunderstood by friends, echoing experiences documented in other chronic illness contexts [6, 9, 34, 35, 37, 38]. Being socially isolated was likened to feeling like a *dead tree*, symbolising a lack of vitality and connection with others. Parents also echoed these social challenges, expressing emotional distress about their children missing out on normal social experiences and the difficulties in supporting their independence. As other studies have noted, the unpredictability, variability and lack of formalised information and support complicated parents’ efforts to support their children to have a ‘normal’ social life [39].

The self-management strategies employed by CYP and parents, such as energy conservation techniques and lifestyle adjustments, underscored their proactive approach to coping with SCD fatigue in the absence of formalised support. These strategies mirror those reported in studies of adolescent cancer survivors and other individuals with SCD, emphasising the importance of pacing and prioritisation in managing daily activities [26, 36, 40, 41]. Being able to economise their reduced energy capacity efficiently meant CYP needed to understand their bodies and personal limits, constantly weighing the cost and benefits of activities and being able to discipline and regulate themselves strictly. However, this was not always realistic for the CYP due to a lack of understanding, acceptance and support within their social domains coupled with the wider demands of excellence and competence. As they often told us, being the responsible and disciplined CYP who rests, paces themselves and avoids overexertion to self-manage fatigue was in tension with being productive and youthful. This aligns with another qualitative study, where young people with SCD in the UK were conflicted in juxtaposing self-disciplined with self-actualised/productive identities [36]. This forced the CYP to push themselves constantly. Pushing yourself was considered a positive (and inevitable) self-management strategy [40]. This was an important strategy to meet education and social responsibilities, avoid stigmatising social responses, as well as helping the CYP handle social discourses surrounding being young, productive, responsible and self-sufficient, irrespective of the dire consequences for their health [28, 36].

One significant finding pertains to the future implications of SCD fatigue, which pose substantial challenges to CYP’s personal, social and economic development. Fatigue not only compromises their current educational and social participation and achievements but also influences their career aspirations, forcing some young people to rethink their vocational and professional choices to align them with their reduced energy capacity. This adaptation reflected a strategic and resilient response to mitigate the impact of fatigue on their professional prospects and long-term career trajectories in order to work towards becoming neoliberal citizens– responsible, productive, competent and autonomous [26, 28, 36]. There was a palpable sense of fear, uncertainty and anxiety among the CYP, parents and healthcare professionals regarding the future implications of SCD fatigue, such as the ability of the young people to manage family responsibilities, sustain meaningful employment, achieve economic independence and avoid dependence on healthcare services. Seeing their children juggle the immediate consequences of fatigue and its future projections heightened parents’ concerns and anxieties. The lack of recognition and management of fatigue within clinical care also exacerbated these fears as CYP and parents grappled with uncertainties about long-term outcomes and quality of life.

### Implications for services, care and research

This study highlights the multidimensional impact of SCD-related fatigue on young people’s lives, shedding light on its pervasive nature, social and educational consequences, and implications for their future development. It underscores the urgent need for healthcare professionals to systematically recognise and address fatigue within clinical care to empower CYP and their families to manage fatigue effectively and enhance their overall quality of life. Our research participants indicate that fatigue should be integral to SCD care and included in (a) health assessments (e.g., annual reviews, psychological assessments, admission, and discharge plans), (b) patient and parent education programmes, (c) healthcare professional education and training, (d) school education and care plans, and (e) public education and SCD advocacy. Study participants emphasised the importance of improving knowledge and understanding of SCD-related fatigue among care providers, schools, employers, third-sector organisations, and the wider society. This awareness is crucial in raising the profile of the symptom and supporting CYP with SCD.

Our research has highlighted the limited understanding and resources available to identify and manage SCD-related fatigue. Consequently, addressing fatigue should be a research priority in SCD. Research on cancer-related fatigue suggests that a systematic approach is necessary for understanding this phenomenon in SCD across the lifespan. As noted by several researchers [12, 42, 43], more research, including longitudinal studies, is required to fully quantify the burden of fatigue in CYP living with SCD. There is a need to develop treatment pathways for fatigue that incorporate assessment tools, treatment, and self-management interventions/technologies. Therefore, future research should (a) examine factors that contribute to or exacerbate fatigue in CYP to identify specific causal mechanisms, (b) optimise existing patient-reported outcome measurement tools for SCD-related fatigue, (c) assess fatigue as an endpoint for existing and future disease-modifying therapies and self-management interventions/technologies, and (d) develop self-management tools and programmes that specifically address fatigue. In addition, our research has highlighted the emotional challenges linked to fatigue and a more targeted exploration of fatigue’s emotional consequences is needed. Future research on SCD fatigue should consider targeted recruitment strategies to include non-African/Caribbean heritage participants and different settings/contexts, e.g., SCD families of South Asian, Middle Eastern, Mediterranean and mixed heritage and migrants, refugees and asylum-seekers. Such efforts could help broaden our understanding of SCD fatigue and its intersection with diverse cultural, social and healthcare contexts.

It is essential to develop policy frameworks that acknowledge fatigue as a key aspect of SCD care and incorporate it into both national and local health policies to bridge existing gaps in understanding and managing SCD-related fatigue. Policies should support the inclusion of fatigue support in healthcare practices, encourage collaboration across education, employment, and third-sector organisations to promote accommodations and community resources (e.g., advocacy) and allocate funding for research, clinical advancements, and public education on SCD fatigue.

### Strengths and limitations

This study’s strength lies in the diversity of its sample, which included CYP, parents and healthcare professionals from various disciplines and locations across England. Another key strength is the involvement of three young people with SCD in designing and conducting the study and disseminating the findings. They participated in all stages of the research, from developing the initial research idea to ensuring the study’s relevance and meaningfulness to CYP and their families by providing diverse perspectives. Additionally, using art-based approaches enhanced the comprehensiveness of the data collected, supporting the young participants in sharing their experiences more effectively. However, there are some limitations. The interviews were conducted at a single time point, so the findings do not account for changes in fatigue or its management over time and at different stages of a child’s development. Additionally, excluding children younger than 12 years and lacking participants from non-African/Caribbean backgrounds may be considered limitations. Fewer male CYP were interviewed than female CYP; however, the in-depth interviews generated rich data from both sexes.

## Conclusion

This study reveals the significant impact of SCD fatigue on CYP, affecting their physical, social and educational lives. Often misunderstood by others, SCD fatigue leads to isolation and stigma. It is frequently overlooked in clinical settings due to a lack of standardised assessment tools. Addressing fatigue should be integral to SCD care, including health assessments, education programmes and public advocacy. Future research should focus on factors contributing to fatigue, optimising measurement tools, developing self-management programmes and including fatigue as an outcome in treatment evaluations. A comprehensive approach will improve support and quality of life for CYP with SCD. These insights also have broader implications for all CYP dealing with fatigue, highlighting the need for a comprehensive approach across various LTCs, with fatigue addressed as a significant symptom rather than a secondary concern.

## Data Availability

The data generated and/or analysed during the current study are not publicly available due to privacy or ethical considerations. The point of contact regarding the availability of data and materials is the corresponding author.

## Abbreviations

CYP: Children and Young People
HCP: Health Care Professional
LTC: Long-Term Condition
P: Parent
SCD: Sickle Cell Disease

## Footnotes

1 The terms ‘children’ and ‘young people’ have been used as defined by the United Nations. Young people are defined as those aged 15–24, and children are those younger than 18 (https://www.un.org/en/global-issues/youth). We have used the term ‘children and young people’ as a collective term for those younger than 25 years.

2 Participants were identified by their unique identifier, plus sex and age for the CYP participants (e.g., CYP08, male, 12 years = CYP participant 8) or plus child’s sex and age for parent participants (P01, male child, 14 years = Parent 1), or plus disciplinary background for healthcare professional (HCP01, specialist nurse = Healthcare Professional participant 1).
